# Comparison of the Effects of TIPS versus BRTO on Bleeding Gastric Varices: A Meta-Analysis

**DOI:** 10.1155/2020/5143013

**Published:** 2020-02-11

**Authors:** Zi Wen Wang, Jin Chao Liu, Fang Zhao, Wen Guang Zhang, Xu Hua Duan, Peng Fei Chen, Si Fu Yang, Hong Wei Li, Fu Wen Chen, Hong Sheng Shi, Jian Zhuang Ren

**Affiliations:** ^1^Radiology Intervention Department, Puyang Oilfield General Hospital, Puyang, China; ^2^Radiology Intervention Department, First Affiliated Hospital of Zhengzhou University, Zhengzhou, China

## Abstract

*Background and Aim*. Upper gastrointestinal bleeding is a threat to patients with gastric varices (GVs). Previous studies have concluded that both transjugular intrahepatic portosystemic shunt (TIPS) and balloon-occluded retrograde transvenous obliteration (BRTO) are effective treatments for patients with GV. We aimed to compare the efficiency and outcomes of these two procedures in GV patients through meta-analysis.

**Methods:**

The PubMed, Cochrane Library, EMBASE, and Web of Science databases were searched using the keywords: GV, bleeding, TIPS, and BRTO to identify relevant randomized controlled trials and cohort studies. The overall survival (OS) rate, imminent haemostasis rate, rebleeding rate, technical success rate, procedure complication rate (hepatic encephalopathy and aggravated ascites), and Child-Pugh score were evaluated. Randomized clinical trials and cohort studies comparing TIPS and BRTO for GV due to portal hypertension were included in our meta-analysis. Two independent reviewers performed data extraction and assessed the study quality. A meta-analysis was performed to calculate risk ratios (RRs), mean differences (MDs), and 95% CIs using random effects models.

**Results:**

A total of nine studies fulfilled the inclusion criteria. There was a significant difference between TIPS and BRTO in the OS rate (RR, 0.81 (95% CI, 0.66 to 0.98); *P*=0.03) and rebleeding rate (RR, 2.61 (95% CI, 1.75 to 3.90); *P*=0.03) and rebleeding rate (RR, 2.61 (95% CI, 1.75 to 3.90); *P*=0.03) and rebleeding rate (RR, 2.61 (95% CI, 1.75 to 3.90); *P*=0.03) and rebleeding rate (RR, 2.61 (95% CI, 1.75 to 3.90); *P*=0.03) and rebleeding rate (RR, 2.61 (95% CI, 1.75 to 3.90); *P*=0.03) and rebleeding rate (RR, 2.61 (95% CI, 1.75 to 3.90); *P*=0.03) and rebleeding rate (RR, 2.61 (95% CI, 1.75 to 3.90);

**Conclusions:**

In this meta-analysis, BRTO brought more benefits to patients, with a higher OS rate and lower rebleeding rate. BRTO is a feasible method for GVB.

## 1. Introduction

Gastric varices (GVs) are a common complication associated with portal hypertension [1]. Previous studies [2–6] have found that gastric varices bleeding (GVB) is lower in prevalence compared with oesophageal varices bleeding (EVB) but is associated with considerably higher mortality (45–55%) and rebleeding (35–90%) rates.

The current treatment approaches for GVB caused by portal hypertension include endoscopic therapy, radiological intervention methods, and surgical management. Endoscopic therapies such as endoscopic variceal ligation (EVL) and endoscopic variceal obturation (EVO) are considered effective methods for treating GVB [7]. However, EVL and EVO are limited in value for GV bleeding. Current guidelines [8, 9] suggest that EVL should be performed on GVs smaller than 2 cm in diameter because ligation can be performed with standard rubber bands, whereas larger-diameter GVs require the use of larger detachable snares, which can increase the risk of rebleeding [10]. EVO is considered a more effective method of haemostasis [11, 12], but the complications of EVO and thromboembolic phenomena (splenic, renal, pulmonary, cerebral, spinal, and coronary) may be serious and even life-threatening.

Radiological intervention methods also have demonstrated efficiency and safety in managing GVB [13–16]. Transjugular intrahepatic portosystemic shunt (TIPS), a minimally-invasive interventional surgery, can decompress the portal circulation and is regarded as the primary approach for GVB management in the West (United States and Europe) [17]. TIPS reduces GVB and treats complications with liver cirrhosis, such as refractory ascites and hepatic encephalopathy. Balloon-occluded retrograde transvenous obliteration (BRTO) is also a suitable method for GVB because it scleroses the GV, and it is widely used in the East (Japan and Korea) [18]. However, the portal pressure may not decrease due to a lack of shunts, which can cause complications such as ascites and worsening of oesophageal varix pressure. With the development of radiation intervention techniques, TIPS and BRTO have been increasingly widely used in both Western and Eastern countries. The current studies [19–22] on TIPS and BRTO have shown that both methods can be beneficial for reducing the rebleeding rate and improving the overall survival rate. However, whether TIPS or BRTO is more beneficial for GVB patients, especially regarding the overall survival rate, is still unknown. We searched databases and found a meta-analysis [23] including five studies to compare the effects of TIPS and BRTO. Three of the studies were abstracts without the full article [24–26], which may have caused incomplete data bias. Therefore, we performed a systematic review and meta-analysis to evaluate the feasibility and safety of TIPS and BRTO.

## 2. Methods

This meta-analysis was performed according to the Cochrane Handbook for Systematic Reviews of Interventions and is presented based on the Preferred Reporting Items for Systematic Reviews and Meta-Analyses guidelines.

### 2.1. Search Trials

We searched the PubMed, Cochrane Library, EMBASE, and Web of Science databases from their inception dates to October 10, 2019, using the keywords gastric varices, bleeding, transjugular intrahepatic portosystemic shunt (TIPS), and balloon-occluded retrograde transvenous obliteration (BRTO), to identify relevant published randomized controlled trials and cohort studies. There were no language restrictions. We excluded trials and cohort studies that included only TIPS or BRTO without comparison.

### 2.2. Inclusion Criteria

Trials were selected based on the following inclusion criteria: (1) RCTs and cohort studies comparing TIPS and BRTO; (2) patients with a clear diagnosis of GVs due to portal hypertension; and (3) studies providing original data on patients' baseline characteristics and postprocedure outcomes (postprocedure overall survival rate at five years, immediate haemostasis rate, rebleeding rate, technical success rate, procedure complication rate, and Child-Pugh score). The exclusion criteria were as follows: (1) randomized trials or cohort studies only about TIPS or BRTO without comparison; (2) case reports, reviews, meta-analyses, and studies or trials without specific dates on patient enrolment.

### 2.3. Risk-of-Bias Assessments

The methodological quality of the included RCTs was assessed independently by two researchers (Z. W. W. and P. F. C.) based on the Cochrane risk-of-bias criteria. Cohort studies were evaluated using the Newcastle–Ottawa Scale (NOS). In brief, a maximum of 9 points was assigned to each study: 4 for selection, 2 for comparability, and 3 for outcomes. Studies with a final score ≥6 were regarded as high quality. The rankings for each study are shown in Table 1.

### 2.4. Data Extraction

Two researchers (Z. W. W. and P. F. C.) independently extracted the following information for each study: lead author, publication year, country of origin, patient characteristics, overall survival rate, immediate haemostasis rate, rebleeding rate, technical success rate, procedure complication rate (hepatic encephalopathy and aggravated ascites), and Child-Pugh score. We extracted only the information and data of interest reported in the original articles. If a meta-analysis noted that unpublished data were provided by the primary authors, we extracted those missing data the from forest plots of the meta-analysis and reviewed the original articles to confirm whether the trials met our inclusion criteria. When those data were our outcomes of interest, we pooled them with the data from the primary trials.

The postprocedure overall survival rate was the primary outcome of interest because it represented the most direct benefit to patients. The secondary outcomes of interest were the number of patients with rebleeding after procedure, immediate haemostasis, technical success, and procedure complications as well as the Child-Pugh score. If a trial or study only reported the baseline Child-Pugh score or Child-Pugh grade without the score (Child-Pugh A, B, C), we did not consider those to be valid data.

### 2.5. Statistical Analysis

We performed a meta-analysis to calculate risk ratios (RRs), mean differences (MDs), and 95% confidence intervals (CIs) using the Mantel–Haenszel statistical method. If zero events were reported for one group in a comparison, a value of 0.5 was added to both groups for each such study. Based on the recommendation of the Cochrane Handbook, trials or studies with zero events in both the intervention and the control groups were not included in the meta-analysis when RRs were calculated.

A random effects model was used to pool the data, and statistical heterogeneity between summary data was evaluated using the *χ*^2^ test and *I*^2^ statistic. Sensitivity analysis was performed by excluding low-quality studies or trials with characteristics different from the others. If possible, potential publication bias was assessed by a visual inspection of the funnel plots of the primary outcome.

All meta-analyses were performed using RevMan version 5.3 (Cochrane Collaboration). All tests were 2-tailed, and *P* < 0.05 was considered statistically significant.

## 3. Results

### 3.1. Studies Retrieved and Characteristics

**Figure 1:**
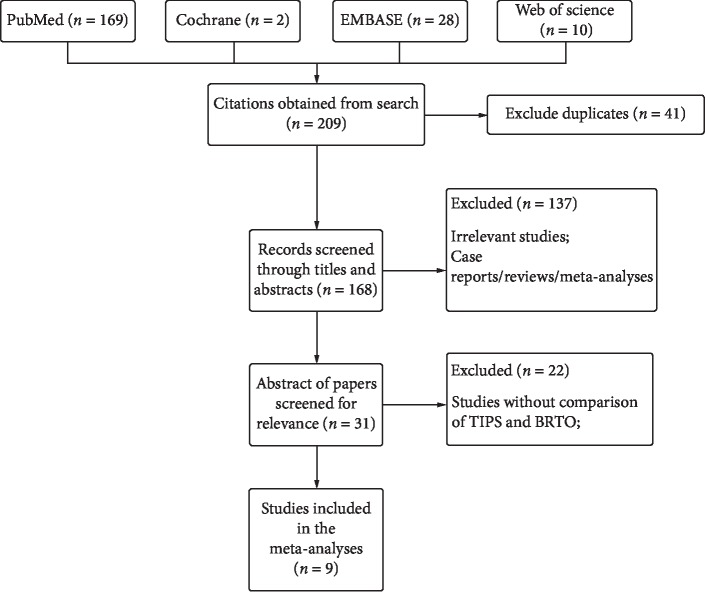
Literature search and screening process.

Based on the database search strategy, a total of 209 articles were identified. The titles and abstracts of these records were screened for inclusion. Forty-one articles were removed after finding duplicates. The full texts of thirty-one records were read, and nine [24–32] met the inclusion criteria (Figure 1): one RCT and eight cohort studies. The specific details of the papers that were included in our study are displayed in Table 2. The quality of the RCT was evaluated by the Cochrane ROB (good), and the quality of the cohort studies was evaluated by the Newcastle–Ottawa Scale with a final score ≥6 points, which means that all these studies had considerable methodological quality.

### 3.2. Meta-Analysis of the Overall Survival Rate

**Figure 2:**
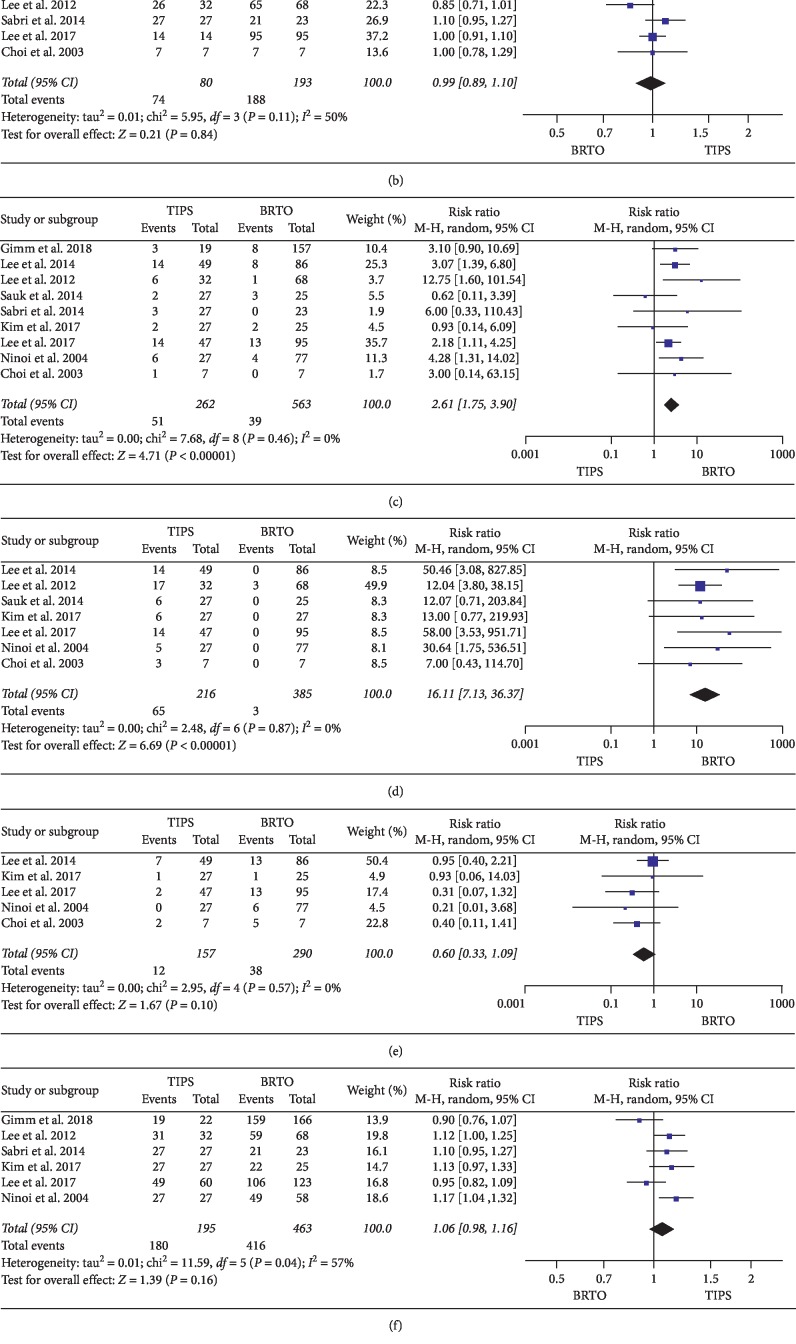
Meta-analysis results: (a) overall survival rate, (b) haemostasis rate, (c) rebleeding rate, (d) hepatic encephalopathy rate, (e) aggravated ascites rate, (f) technical success rate, and (g) Child-Pugh change. There was a significant difference between TIPS and BRTO in the overall survival rate (RR, 0.81 (95% CI, 0.66 to 0.98); *P*=0.03) and rebleeding rate (RR, 2.61 (95% CI, 1.75 to 3.90); *P* < 0.00001). TIPS had a higher incidence rate of hepatic encephalopathy (RR, 16.11 (95% CI, 7.13 to 36.37); *P* < 0.00001). There was no significant difference between TIPS and BRTO in the immediate haemostasis rate (RR, 0.99 (95% CI, 0.89 to 1.10); *P*=0.84), technical success rate (RR, 1.06 (95% CI, 0.98 to 1.16); *P*=0.16), aggravated ascites rate (RR, 0.60 (95% CI, 0.33 to 1.09); *P*=0.10), or Child-Pugh change (MD, 0.22 (95% CI, −0.21 to 0.65); *P*=0.31).

Five studies comparing the OS rates between TIPS and BRTO demonstrated that the OS rate in the BRTO group was higher than that in the TIPS group. There was a significant difference between the two groups (RR, 0.81 (95% CI, 0.66 to 0.98); *P*=0.03) according to a random effects model with no heterogeneity (*P*=0.14; *Ι*^2^ = 40%) (Figure 2).

### 3.3. Meta-Analysis of the Rebleeding Rate

All nine studies compared the postprocedure rebleeding rates between TIPS and BRTO. The rebleeding rates in the TIPS groups was higher than that in the BRTO group. There was a significant difference between the two groups (RR, 2.61 (95% CI, 1.75 to 3.90); *P* < 0.00001) according to a random effects model with no heterogeneity (*P*=0.46; *Ι*^2^ = 0%) (Figure 2).

### 3.4. Meta-Analysis of Postprocedure Complications

Four studies compared the postprocedure complication rate between TIPS and BRTO. The hepatic encephalopathy rate after the procedure in the TIPS group was significantly higher than that in the BRTO group (RR, 16.11 (95% CI, 7.13 to 36.37); *P* < 0.00001) under a random effects model, with no heterogeneity (*P*=0.87; *Ι*^2^ = 0%). The incidence rate of aggravated ascites in the BRTO group was higher than that in the TIPS group without a significant difference (RR, 0.60 (95% CI, 0.33 to 1.09); *P*=0.10) according to a random effects model with no heterogeneity (*P*=0.57; *Ι*^2^ = 0%) (Figure 2).

### 3.5. Meta-Analysis of the Immediate Haemostasis Rate

Three studies compared the postprocedure immediate haemostasis rate between TIPS and BRTO. The postprocedure immediate haemostasis rate in the TIPS group was higher than that in the BRTO group. Nevertheless, there was no significant difference between the two groups (RR, 0.99 (95% CI, 0.89 to 1.10); *P*=0.84) according to a random effects model with no heterogeneity (*P*=0.11; *Ι*^2^ = 50%) (Figure 2).

### 3.6. Meta-Analysis of the Technical Success Rate

Five studies compared the technical success rate between TIPS and BRTO. The technical success rate in the TIPS group was higher than that in the BRTO group. Even so, there was no significant difference between the two groups (RR, 1.06 (95% CI, 0.98 to 1.16); *P*=0.16) according to a random effects model with heterogeneity (*P*=0.04; *Ι*^2^ = 57%) (Figure 2).

### 3.7. Meta-Analysis of Changes in Child-Pugh Score

Three studies compared the changes in the Child-Pugh score between TIPS and BRTO. The change in the Child-Pugh score in the BRTO group was higher than that in the TIPS group, but this difference did not reach statistical significance (MD, 0.22 (95% CI, −0.21 to 0.65); *P*=0.31) according to a random effects model with no heterogeneity (*P*=0.32; *Ι*^2^ = 13%) (Figure 2).

## 4. Discussion

The management of GVB is not incontrovertible and poses substantial clinical challenges. Given the limited efficacy of endoscopic treatment and candidacy restrictions of shunt surgery, endovascular treatments such as BRTO and TIPS have become valuable options for controlling GVB [34]. Therefore, the discussion of the choice of these two surgical methods is ongoing. The two treatments are completely different in their therapeutic concepts: TIPS aims to decrease the portal pressure to reduce gastric variceal bleeding [31], and BRTO scleroses the GV without decreasing portal pressure [33]. TIPS changes the haemodynamics of the portal vein: the blood flow from the portal vein into the inferior vena cava, which reduces the blood supply to the liver and increases the burden on the liver. On the contrary, some blood that has not been metabolized by the liver directly enters the systemic circulation and might lead to azotaemia, which in turn leads to hepatic encephalopathy and hepatic myelopathy. BRTO directly stops bleeding without reducing portal pressure, which may increase the risk of ascites and bacterial peritonitis. The long-term effects of BRTO on portal haemodynamics have been reported [35, 36]. Using BRTO for treating GV, as with the TIPS procedure, may preserve the function and synthetic capabilities of the liver. We pooled three studies to compare the changes in the Child-Pugh score after the procedure to evaluate liver function. The meta-analysis showed that there was no significant difference between the two groups in the Child-Pugh score change.

For patients, a long survival time is the most valuable benefit. Gimm et al. reported that the postprocedure OS after 5 years was longer after BRTO than after TIPS [27]. Ninoi et al. reported that among patients in Child-Pugh class A, survival (5 years) was significantly higher in the transcatheter sclerotherapy group (including BRTO) than in the TIPS group. In Child-Pugh classes B and C, no significant difference was seen between the TIPS and transcatheter sclerotherapy groups [31]. Lee et al. reported that the postprocedure OS after three years was longer after BRTO than after TIPS [29]. Choi et al. and Sabri et al. reported that the postprocedure OS after one year was higher in BRTO than in TIPS [30, 32]. Kim et al. reported that there was no statistically significant difference in the mean survival between the two groups (TIPS, 30 months; BRTO, 24 months; *P*=0.16), but the median survival of the patients who underwent TIPS was 16.6 months and that for patients who underwent BRTO was 26.6 months [28]. Therefore, we compared the OS rates between TIPS and BRTO. The combined data suggested that the BRTO group had a significantly higher OS rate than the TIPS group. Early TIPS treatment is still the first-line treatment for acute upper gastrointestinal bleeding. García-Pagán et al. [37] reported that the early use of TIPS was associated with a reduction in mortality. Qi et al. performed a meta-analysis and demonstrated that compared with medical/endoscopic therapy for acute variceal bleeding, TIPS with covered stents might improve overall survival [38]. Nevertheless, there were some limitations in that meta-analysis: (a) only 3 of 6 studies discussed TIPS for GV, and (b) the meta-analysis only compared medical/endoscopic therapy with TIPS for acute variceal bleeding, BRTO was not mentioned. In our study, we compared TIPS and BRTO for GV. The overall survival rate and rebleeding rate in the BRTO group were significantly higher than those in the TIPS group. This finding may provide new options for the future treatment of GV.

The technical difficulty of TIPS lies in portal vein puncture: puncture may cause massive haemorrhage in the abdominal cavity. The appropriate positioning of the puncture point may lead to shunt dysfunction, which makes it difficult to reduce portal pressure [39, 40]. BRTO is also a relatively demanding interventional surgery that requires a well-knit and skilled basis in anatomy, radiology, and handling procedures. Wang et al. reported that BRTO was not more difficult to finish than TIPS [23]. In our meta-analysis, we reached a similar conclusion. The rebleeding rate of the TIPS group was significantly higher than that in the BRTO group. As previously shown, especially in shunts with bare metal stents, thrombosis could occur in the shunt, which leads to an increase in the rebleeding rate [41]. Qi et al. performed a meta-analysis and demonstrated that compared with bare stents, covered stents for TIPS may improve overall survival. Subgroup analysis for the effect of different brands of covered stents achieved no significant difference [42]. In our meta-analysis, there were only two early studies using bare stents [31, 32]. The original data of all the studies did not specifically record the postoperative changes in patients with bare or covered stents. We tried to exclude the early research that contained bare metal stents [31, 32] before analysing the data and reached the same conclusion on the rebleeding rate. Tripathi et al. and Chau et al. showed that GV can bleed at PSGs <12 mmHg and behave differently than EVs, which bleed at higher PSGs (average, 15.5 mmHg) [43, 44]. Thus, even though the portal shunt was established, PSGs might not decrease below 12 mmHg. Another meta-analysis suggested that TIPS in combination with variceal embolization is more effective for the prevention of variceal rebleeding [45]. In our included studies, a total of 23 patients underwent post-TIPS coil embolization. In the study by Sabri et al. [30], if any GV were visualized during the TIPS procedure, selective catheterization of the inflow portal venous branch was followed by embolization of the branch using metallic coils. In the study by Choi et al. [32] and Kim et al. [28], gastric varices were evaluated by CT after the TIPS procedure. If the GV persisted, additional embolization was performed. However, in the results analysis section, the above authors did not list the data on patients with TIPS combined with variceal embolization separately. We were unable to obtain raw data from these studies and to use these data for further analysis of whether a combination of TIPS with variceal embolization is superior to BRTO alone for GV. More RCTs are needed in the future. Except for the postprocedure complications (hepatic encephalopathy), the TIPS group and BRTO group did not have a significant difference in the immediate haemostasis rate, which meant that the two methods were both effective for GVB in emergency situations.

Our meta-analysis only contained one randomized controlled trial with a small sample size, which may have led to selection bias. Differences in the performance of different TIPS stents might have also affected the results. With the development of radiological intervention technology, more RCTs comparing TIPS and BRTO should be conducted.

## 5. Conclusions

In our meta-analysis, BRTO brought more benefits to patients than TIPS. BRTO is a suitable method for GVB with gastrorenal shunts.

## Figures and Tables

**Table 1:** Risk-of-bias assessment of the included studies.

No.	Study ID	Newcastle–Ottawa quality assessment scale for cohort studies									
		Selection				Comparability		Outcome			Total score
		1	2	3	4	5	6	7	8	9	
1	Gimm et al. [27]	★	★	★	●	★	●	★	★	★	7
2	Kim et al. [28]	★	★	★	●	★	★	★	★	★	8
3	Lee et al. [29]	★	★	★	●	★	★	★	★	★	8
4	Sabri et al. [30]	★	★	★	●	★	★	★	★	●	7
5	Lee et al. [24]	★	★	★	●	★	●	★	★	●	6
6	Sauk et al. [26]	★	★	★	●	★	●	★	★	●	6
7	Lee et al. [25]	★	★	★	●	★	●	★	★	●	6
8	Ninoi et al. [31]	★	★	★	●	★	★	★	★	★	8

1: representativeness of the exposed cohort; 2: selection of the nonexposed cohort; 3: ascertainment of exposure; 4: demonstration that the outcome of interest was not present at the start of the study; 5: study controls for the most important factor; 6: study controls for the second-most important factor; 7: assessment of outcome; 8: follow-up long enough for outcomes to occur; 9: adequacy of follow-up of cohorts. ★ was awarded if the respective information was available. ● was awarded if the respective information was unavailable.

**Table 2:** Characteristics of the included RCT and cohort studies.

Included studies	Country	Design	Sample size			Women, *n* (%)	Mean age		Follow-up	Traits of patients	Type of TIPS stents	Outcomes
			Total	TIPS	BRTO		TIPS	BRTO				
Gimm et al. [27]	Korea	Cohort	176	19	157	37 (21)	59.4	54.4	5 years	GV bleeding	Covered	IM, RB, C, OS
Kim et al. [28]	Korea	Cohort	52	27	25	24 (46)	58.0	59.0	1 year	GV bleeding (isolated GV)	Covered	TS, C, MELD score, IM, RB, OS
Lee et al. [29]	Korea	Cohort	142	47	95	27 (19)	55.6	59.4	3 years	GV bleeding	Covered	TS, Child-Pugh, C, RB, IM, OS
Sabri et al. [30]	USA	Cohort	50	27	23	21 (42)	55.0	52.0	1 year^▲^	GV bleeding (isolated GV)	Covered	TS, C, OS, RB, H
Lee et al. [24]	Korea	Cohort	135	49	86	—	58.5	58.5	2 years	GV bleeding	—	RB, OS, C,
Sauk et al. [26]	USA	Cohort	52	27	25	24 (46)	58.0	59.0	—	GV bleeding (isolated GV)	—	C, RB,
Lee et al. [25]	Korea	Cohort	100	32	68	—	—	—	—	GV bleeding	—	TS, RB, C, OS
Ninoi et al. [31]^◆^	Japan	Cohort	76	27	58	34 (58)	58.7	60.3	5 years^*∗*^	GV bleeding	Bare	TS, OS, Child-Pugh, C, RB, H
Choi et al. [33]	Korea	RCT	14	7	7	4 (29)	54.0	60.3	1 year^■^	GV bleeding	Bare/covered	OS, Child-Pugh, C, RB, IM

OS: overall survival; PSG: portosystemic gradient; GV: gastric variceal; IM: immediate haemostasis; RB: rebleeding; C: complications; TS: technical success; H:haemostasis. ^◆^Ninoi et al. compared TIPS with transcatheter sclerotherapy (including BRTO). We extracted relevant data about BRTO from the article. ^▲^The mean duration of follow-up was 19.5 months for patients in the TIPS group (range, 1–52 mo.) and 18.2 months for patients in the BRTO group (range, 1–49 mo.). ^*∗*^The follow-up period after the procedures was 41.2 ± 32.4 months in the TIPS group and 26.9 ± 16.5 months in the transcatheter sclerotherapy group (including BRTO). ^■^Patients were followed up for 6 to 21 (mean, 14.4) months. _Not mentioned.
